# Pathology-Driven Genomic Panels for Personalized Prognostic Stratification and Exploratory Therapeutic Prediction in Clear-Cell Renal Cell Carcinoma with Tumor Thrombus

**DOI:** 10.3390/diagnostics16070989

**Published:** 2026-03-25

**Authors:** Chenghao Tan, Shiming He, Sainan Zhu, Qun He, Zhisong He, Liqun Zhou, Gengyan Xiong

**Affiliations:** 1 Department of Urology, Peking University First Hospital, Beijing 100034, China; 2 Department of Biostatistics, Peking University First Hospital, Beijing 100034, China; 3 Department of Urological Pathology, Peking University First Hospital, Beijing 100034, China

**Keywords:** clear-cell renal cell carcinoma, molecular pathology, personalized oncology, multigene panel, companion diagnostics, prognostic biomarker, immunotherapy response

## Abstract

**Background/Objectives**: Traditional histopathologic grading of renal cell carcinoma (RCC) is subjective, is poorly reproducible, and fails to predict responses to modern targeted agents or immunotherapies. In the era of precision oncology, molecular pathology offers objective tools for individualized management. We aimed to characterize genomic alterations in clear-cell RCC (ccRCC) with venous tumor thrombus and to develop pathology-driven panels for personalized prognostic stratification, with exploratory assessment of their potential to predict therapeutic response. **Methods**: Formalin-fixed paraffin-embedded pT1 ccRCC samples with and without thrombus underwent whole-exome sequencing. Distinct somatic mutations and copy number variations were incorporated into multigene panels. External assessment was performed in TCGA and PAWG cohorts, assessing survival outcomes and therapeutic biomarkers including homologous recombination deficiency (HRD), tumor mutational burden (TMB), and microsatellite instability (MSI). **Results**: Thrombus cases showed unique genomic heterogeneity compared with matched controls. Three multigene panels were constructed. Across external datasets, including a 354-patient TCGA-KIRC ccRCC cohort, the panels provided consistent molecular stratification signals for overall, disease-specific, and progression-free survival, complementing established pathological risk factors. They were significantly associated with established therapy-related genomic biomarkers, including HRD, TMB, and MSI, showing high sensitivity and negative predictive value in identifying patients unlikely to harbor these biomarker-positive profiles. These findings support the panels’ prognostic utility, with exploratory evidence for their potential in therapy response prediction. **Conclusions**: Small ccRCC with thrombus harbors distinct molecular pathological features. The proposed pathology-driven panels, compatible with FFPE tissue, represent pathology-compatible genomic tools that may support modern precision pathology by improving molecular risk stratification and informing exploratory therapeutic biomarker assessment.

## 1. Introduction

Renal cell carcinoma (RCC) is among the most frequently diagnosed urological malignancies, ranking sixth in men and eighth in women worldwide [1]. Clear-cell RCC (ccRCC) accounts for the majority of cases and typically exhibits more aggressive clinical behavior compared with papillary or chromophobe subtypes [2,3,4]. Traditionally, the Fuhrman grading system has been widely applied as the key histopathologic index to evaluate tumor aggressiveness [5]. However, interobserver variability significantly limits its reproducibility; in a multicenter study, only 24% of cases were assigned to the same grade by three senior pathologists [6]. These limitations highlight the urgent need for more objective, reproducible, and biologically informed approaches in pathology.

Modern pathology is increasingly defined by the integration of molecular diagnostics and computational methods into routine histology. In an era of personalized oncology, pathology-driven genomic markers can provide critical information beyond morphology, enabling individualized prognostication and therapeutic decision-making [7,8,9]. In particular, patients with ccRCC and venous tumor thrombus represent a clinically high-risk subgroup, often associated with poor outcomes despite relatively small tumor sizes [10,11]. The rapid development of thrombi in these cases suggests unique genomic alterations that cannot be captured by traditional pathology alone.

To address this gap, we performed whole-exome sequencing (WES) on small-diameter (<7 cm) ccRCC with thrombus and compared them with size- and grade-matched controls. Based on distinct somatic mutations and copy number variations, we constructed pathology-guided multigene panels and validated their prognostic and therapeutic relevance across both ccRCC and pan-cancer cohorts from The Cancer Genome Atlas (TCGA) Pan-Cancer database [11] and Pan-cancer Analysis of Whole Genomes (PAWG) database [12]. These panels aim to provide a molecular pathological framework for modern precision pathology, supporting individualized risk assessment and therapy prediction in clinical practice.

## 2. Materials and Methods

### 2.1. Study Design and Participants

To screen for reliable genomic markers of malignancy, we set the following preconditions in the patient enrolling progress: First, all the enrolled individuals must be pathologically diagnosed ccRCC patients. Second, as mentioned in the Introduction, only small-diameter ccRCC (<7 cm or pT1) patients were enrolled. Additionally, the number of patients in the pT1a (or ≤4 cm) and pT1b (or 4–7 cm) groups should be as consistent as possible. Third, in general, higher-grade renal tumor patients are much more likely to have tumor thrombi, so clear-cell renal cell carcinoma with tumor thrombus (ccRCC-TT) patients at relatively low grades may have more aggressive genomic features that existing pathological examinations cannot detect; the high-grade (G4) patients were excluded in our study. 

### 2.2. Library Preparation and Sequencing

In our study, microdissection technology was used on each formalin-fixed and paraffin-embedded (FFPE) section to ensure sample purity. DNA was extracted using the GeneRead DNA FFPE Kit (Qiagen, Hilden, Germany), following the manufacturer’s instructions. After numbering and anonymization, all DNA samples were submitted to the Novogene Company (Beijing, China) for Illumina sequencing. The detailed sequencing and genome analysis processes have previously been described [13].

### 2.3. Endpoints

The primary endpoint of this study was the prognostic performance of the constructed panels, assessed by overall survival (OS), disease-specific survival (DSS), and progression-free survival (PFS) in both the ccRCC and pan-cancer cohorts.

The secondary (exploratory) endpoint was the predictive association between panel status and systemic therapy-related biomarkers, including homologous recombination deficiency (HRD), tumor mutational burden (TMB), and microsatellite instability (MSI), which reflect potential responsiveness to PARP inhibitors, platinum chemotherapy, and immunotherapy.

### 2.4. Panel Construction, TCGA Verification, and Statistical Analysis

Significantly mutated genes were defined as those showing a higher mutation frequency in the high-risk group, driver genes as functionally reported oncogenic or tumor-suppressive genes in public cancer databases, and predisposing genes as hereditary cancer-related genes identified in the existing literature (Supplementary Material File S1).

Panel 1: Identification of baseline high-risk genomic features. To identify unique genomic characteristics associated with ccRCC malignancy for initial gene signature construction, a stepwise screening strategy was applied. First, key somatic mutations specific to the high-risk ccRCC-TT patients were identified by comparing primary tumors and tumor thrombi with controls. Genes with *p* < 0.05 and q < 0.20 were retained, acknowledging that the small sample size required a moderately relaxed q-value threshold to avoid false negatives; only mutations absent in the control cohort were considered. Second, significant somatic copy number variations (CNVs) enriched in the high-risk group were determined by screening CNV regions with *p* < 0.05 and q < 0.05 in primary tumors and thrombi, while excluding those not significant (q ≥ 0.05) in controls. Finally, all high-risk-specific alterations—including somatic mutations, fusions, and CNVs—were integrated to construct Panel 1 (Supplementary Table S2). Importantly, individual gene-level significance was not interpreted as independent biological evidence, and all candidate alterations were subsequently evaluated only at the multigene panel level through external validation.

Panel 2: Expansion using thrombus-related high-risk genomic events. To broaden the applicability of the gene panel and capture additional thrombus-associated malignant features, all twenty thrombus-related samples (ten primary tumors and ten matched venous tumor thrombi) were jointly analyzed as an expanded high-risk cohort. The same mutation and CNV screening pipeline was applied to identify additional recurrent alterations. As the aim of this study was to identify population-level genomic alterations associated with malignant behavior rather than intra-patient evolutionary differences, the panel construction was based on group-level rather than paired analyses. Primary tumor–thrombus pairs originate from the same patient and are therefore not statistically independent; treating them as separate samples may result in pseudo-enrichment (e.g., alterations in two patients appearing as four samples). To minimize false-positive signals arising from this non-independence, more stringent thresholds were applied (mutations: *p* < 0.0025, q < 0.04; CNVs: *p* < 0.0025, and q < 0.0025). The newly identified cross-patient, thrombus-associated alterations were then integrated with Panel 1 to form Panel 2 (Supplementary Table S3), enabling a more comprehensive representation of biologically aggressive genomic markers.

**Figure 1:**
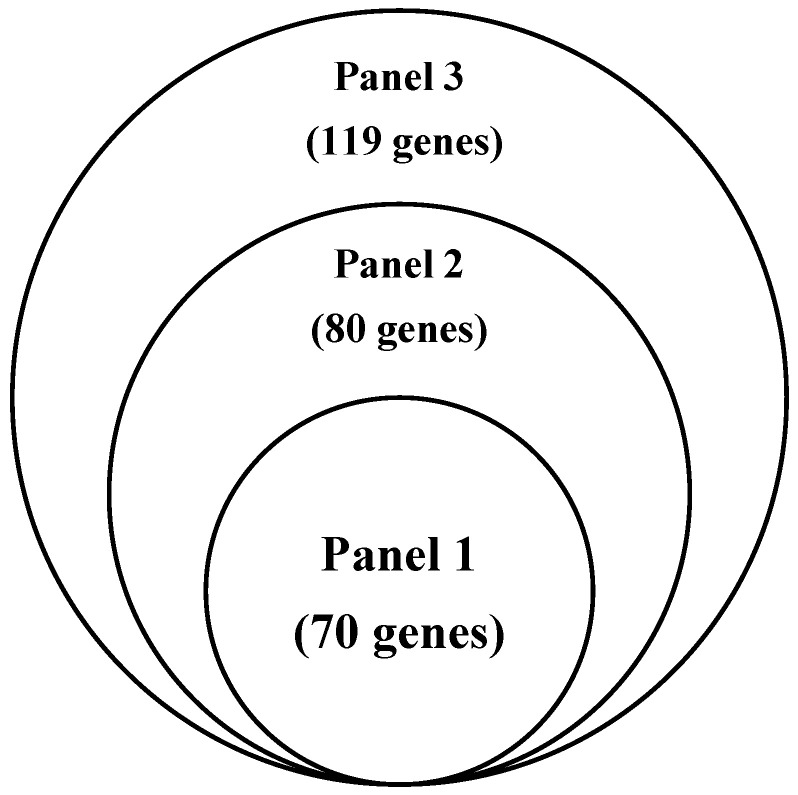
Venn diagram illustrating the overlap and sequential expansion of gene sets across Panels 1–3.

Panel 3: Incorporation of treatment-relevant genomic markers. To further enhance the clinical relevance of the gene signature for treatment guidance, genes related to drug susceptibility were incorporated. Genes in drug-response pathways (Supplementary Figure S1) that were altered in more than half of the high-risk samples—particularly those relating to DOT1L, FLT3, JAK2, and MEK inhibition—were selected, along with genes associated with targeted therapies such as sunitinib, sorafenib [14,15] and mTOR inhibitors [16]. These treatment-relevant genomic markers were combined with Panel 2 to establish Panel 3 (Supplementary Table S4), which provides both prognostic insights and potential utility for the prediction of systemic treatment responsiveness. Gene overlaps across Panels 1–3 are summarized using a Venn diagram (Figure 1).

To verify the reliability of the panels, The Cancer Genome Atlas (TCGA) Pan-Cancer database (non-ccRCC subtypes were excluded) [11] and the Pan-cancer Analysis of Whole Genomes (PAWG) database [12] were applied for dual external assessment, and the grouping process was performed on the cBioPortal platform [17,18]. To ensure clinical interpretability and practical use, the model followed an all-or-none rule: patients were defined as altered if any gene in the panel exhibited a mutation, fusion, or copy number alteration, and as unaltered only if no such alteration was detected in any gene. This dichotomous definition was chosen to enhance interpretability and sensitivity in pathology-oriented applications, rather than to provide a quantitative or weighted assessment of genomic risk. We adhered to this principle through verification throughout the process since the same CNV fragment may cover multiple genes. To facilitate clinical use, we selected representative genes from CNVs for external assessment. If the CNV fragment covered a possible driver gene, the driver gene was preferentially selected; if it did not, a gene was randomly selected (Supplementary Tables S2–S4). As a widely recognized pathology-based prognostic model in renal cell carcinoma, the SSIGN score incorporates tumor stage, tumor size, nuclear grade, and tumor necrosis [19]. However, complete tumor size and necrosis data were not consistently available in the TCGA-KIRC dataset used for external validation. Therefore, a simplified pathology-based baseline model using pT stage and tumor grade was constructed for model comparison.

In statistical analysis, *p*-values for somatic mutations and CNV alterations were derived from group comparisons between the high-risk (thrombus) and control cohorts, and adjusted for multiple testing by false discovery rate (FDR) correction to obtain q-values. Significantly Mutated Genes (SMGs) were identified using the Genome MuSiC package (v0.04), which computes *p*-values and FDRs based on Fisher’s Combined *p*-value Test (FCPT), the Likelihood Ratio Test (LRT), and the Convolution Test (CT). A gene is considered significantly mutated if at least two of the three tests meet the preset FDR threshold. Statistical differences between two groups were analyzed using the Mann–Whitney U test for non-normally distributed continuous variables and ordinal variables (e.g., tumor grade), and the chi-square test for categorical variables. Univariate log-rank tests and multivariate Cox regression were performed to assess the prognostic significance of each variable for DSS, OS, and PFS in the ccRCC population. As malignant tumors usually have certain biological commonalities, we also externally verified the prognostic effects of the panels in the whole pan-cancer population across the two databases. To account for multiple hypothesis testing, different correction strategies were applied according to the statistical context. For survival and external validation analyses (Panels 1–3 across OS, DSS, and PFS), *p*-values were adjusted using the Benjamini–Hochberg false discovery rate (FDR) method. Biomarker comparisons (TMB, MSI, and HRD) were treated as a separate-hypothesis family and similarly adjusted using FDR. For multivariate Cox regression analyses, *p*-values for Panels 1–3 were adjusted using the Holm method within each endpoint–subgroup model. Multiple testing correction was performed separately within each hypothesis family. All statistical analyses were conducted using SPSS (version 29.0, IBM Corp., Armonk, NY, USA) and GraphPad Prism (version 10.0, GraphPad Software, Boston, MA, USA).

## 3. Results

### 3.1. Genomic Characteristics

**Figure 2:**
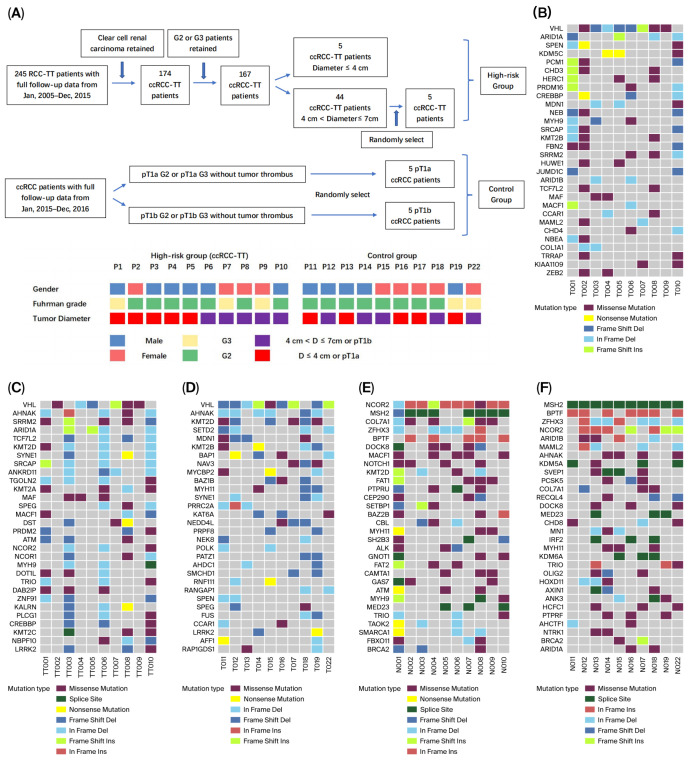
Patient enrolment process, clinical characteristics, driver genes, and predisposing genes. (**A**) Patient enrolment process and clinical characteristics. (**B**) Top thirty driver genes of primary tumors (high-risk group). (**C**) Top thirty driver genes of tumor thrombi (high-risk group). (**D**) Top thirty driver genes in the control group. (**E**) Predisposing genes in the high-risk group. (**F**) Predisposing genes in the control group. Mutation types were summarized by the highest impact alteration per gene per sample. Driver genes in Figure 2 were ranked according to their mutation frequency across samples.

The screening process and general information on enrolled patients, the basic clinical characteristics and sequencing information are summarized in Figure 2A and Supplementary Table S1. With a 162× average sequencing depth, a total of 16,103 non-synonymous somatic changes were identified in our study, including 7165 single-nucleotide variants (SNVs) and 8938 insertions or deletions (InDels).

#### 3.1.1. Significantly Mutated Genes (SMGs) and Drug Susceptibility

The somatic mutations with a false discovery rate (FDR) q-value < 0.2 in each group are shown in Supplementary Tables S5–S7. Ten genes (VHL, FLG2, KRTAP5-7, KRT75, KRTAP10-10, KRTAP6-3, KRTAP10-3, FAM71E2, ANKRD33, and CRIPAK) in the ccRCC-TT primary tumor group, thirteen genes (VHL, KRTAP5-8, PRG4, RPTN, SETD1A, AHNAK2, KRTAP5-11, MAF, KRTAP6-1, FOXB2, FLG2, WBP2NL, and TCHH) in the ccRCC-TT thrombus group, and ten genes (AR, KRTAP5-3, VHL, FLG2, FLG, AHNAK2, KRTAP5-7, MEOX1, RBM14-RBM4, and BAP1) in the control group were identified as SMGs with an FDR q-value < 0.05. The somatic mutations were also compared across four databases (PG_FDA, DrugBank, PharmGKB, and MyCancerGenome) to predict drug susceptibility. The details of the drug effectiveness analysis and the potentially effective drugs affecting more than 40% of the total sample are provided in Supplementary Data S1.

#### 3.1.2. Driver Genes, Predisposing Genes, and Somatic CNVs

**Figure 3:**
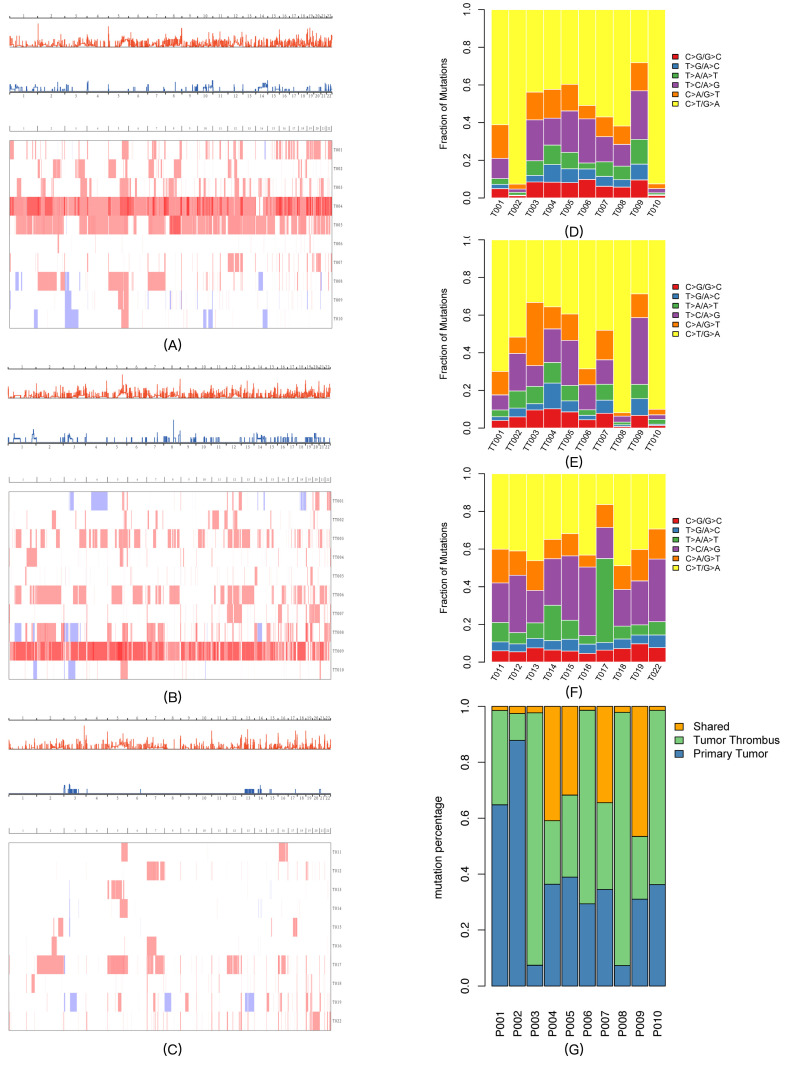
Copy number variation distributions, mutation spectra, and tumor heterogeneity. (**A**) CNV GISTIC curve and heatmap of primary tumors (high-risk group). (**B**) CNV GISTIC curve and heatmap of tumor thrombi (high-risk group). (**C**) CNV GISTIC curve and heatmap of the control group. (**D**) Mutation spectra of primary tumors (high-risk group). (**E**) Mutation spectra of tumor thrombi (high-risk group). (**F**) Mutation spectra of the control group. (**G**) Non-synonymous mutations between the primary tumor and tumor thrombus groups.

The top 30 driver genes in each group are shown in Figure 2B–D. Although VHL was the main driver gene in all three groups, their respective gene compositions were quite different. The top 30 possible predisposing genes in normal samples in the tumor thrombus and control groups are summarized in Figure 2E,F. The somatic CNV frequency distribution and heatmaps in each group are shown in Figure 3A–C (the detailed CNV analysis is shown in Supplementary Data S2). Several somatic CNVs were identified as being significant in all three groups (residual q-value < 0.05), including 4p16.3 gain, 4q35.2 gain, 5p15.33 gain, 6p22.3 gain, 8q24.3 gain, 10q26.3 gain, 12q24.31 gain, 16p13.3 gain, 17q25.3 gain, 18q23 gain, and 19p13.3 gain.

#### 3.1.3. Mutation Spectrum and Signatures of Somatic Mutations

To explore the mutagenesis of the RCC tumor thrombus, we first analyzed the somatic base mutational spectra of all three groups (Figure 3D–F). C>T/G>A was the main mutation type in all three RCC groups. The signatures were identified by the Bioconductor Somatic Signatures package (v2.24.0) according to the non-negative matrix factorization (NMF) algorithm. In each group, three prominent signatures (A, B, and C) were detected (Supplementary Data S3), and the identified signatures were compared to thirty consensus signatures from the Catalog of Somatic Mutations in Cancer (COSMIC) database. However, none of the signatures in our study reached the threshold (cosine similarity > 0.9).

#### 3.1.4. Genomic Comparison of Primary Tumors Between the Thrombus and Control Groups

Patients with tumor thrombi typically present poorer prognoses, suggesting that there should be potential genomic heterogeneity. In this study, the hypothesis was confirmed in the following aspects: (1) As shown in Figure 2 and Supplementary Tables S5–S7, the SMGs and driver genes were different between the two groups. Among all the SMGs (q-value < 0.05) in the respective groups, two genes (KRTAP10-10 and FAM71E2) in the ccRCC-TT primary tumor group were not detected in the control group, and three genes (KRTAP5-3, MEOX1, and RBM14-RBM4) in the control group were not detected in either the ccRCC-TT primary tumor or thrombus groups. (2) Among all the possible driver genes that affected more than 30% of the samples in the ccRCC-TT primary tumor group, four genes (PCM1, CHD3, HERC1, and CREBBP) were not detected in the control group, and three genes (NAV3, MYCBP2, and NEDD4L) from the control group were not detected in either the ccRCC-TT primary tumor or thrombus groups. (3) As shown in Figure 2E,F, the predisposing genes of adjacent tissues between the two groups were different. (4) In the CNV analysis, the overall gene amplification level was higher in the ccRCC-TT group, and the significant CNV segments were different between the two groups (Figure 3 and Supplementary Data S2). (5) As shown in Figure 3D,F, C>T/G>A was the main mutation type in both groups; however, compared with ccRCC-TT primary tumors, the control group presented more T>C/A>G and T>A/A>T mutations.

#### 3.1.5. Genomic Comparison Between the Thrombus Tissue and the Primary Tumor

Although derived from the same patients, there are significant genomic differences between the tumor thrombus and the primary tumor: (1) Even though the anatomical location was very close, the non-synonymous shared mutations (Figure 3G) and mutation spectra (Figure 3D,E) presented genomic heterogeneity between the tumor thrombus and the primary tumor. (2) As shown in Figure 2 and Supplementary Tables S5 and S6, the SMGs and driver genes between the two groups were very different. Among the somatic mutations that were significant (q-value < 0.05) in their respective groups, three genes (KRT75, KRTAP10-10, and KRTAP10-3) in the primary tumor group were not detected in the tumor thrombus group, and two genes (KRTAP5-11 and FOXB2) in the tumor thrombus group were not detected in the primary tumor group. (3) Among all the possible driver genes that affected more than 30% of the samples in each group, five genes (PRDM2, NCOR1, NOCR2, NBPF10, and BCL9) were detected only in the tumor thrombus group. (4) The loss of 8q21.2 (chr8:86393501-86839249) was only significantly identified in the tumor thrombus group (q value = 3.18 × 10^−6^). (5) Through the KEGG enrichment analysis, the VEGF signaling pathway (hsa04370) was only significantly enriched in the thrombus group (Supplementary Data S4), which provided genomic evidence for the higher targeted therapy sensitivity of advanced ccRCC patients. KEGG pathway enrichment analysis was performed using the Genome MuSiC (v0.04) software package based on somatic mutation data and KEGG pathway gene sets. The number of mutated genes in each pathway was counted, and pathway significance was calculated with coverage and gene length correction to reduce false positives. Pathways containing at least one mutated gene were retained, and a background mutation rate was used to evaluate enrichment significance.

### 3.2. Clinical Panels and Verification

#### 3.2.1. Survival Verification in ccRCC Patients

There were 354 patients with complete gene mutation and CNV data in the TCGA kidney renal clear-cell carcinoma (TCGA-KIRC) pan-cancer database, which was used for the external verification of the panels. As shown in Supplementary Table S2, Panel 1 consisted of 25 key genes and 45 DNA amplification segments. In the TCGA-KIRC cohort, patients classified as altered by Panel 1 exhibited worse OS, DSS, and PFS (all q < 0.05) than those without alterations. Panel 2 was constructed by integrating 34 key genes and 46 DNA amplification segments derived from the combined thrombus-related cohort. Consistent with Panel 1, patients in the altered group of Panel 2 also showed poorer OS, DSS, and PFS outcomes (all q < 0.05).

**Figure 4:**
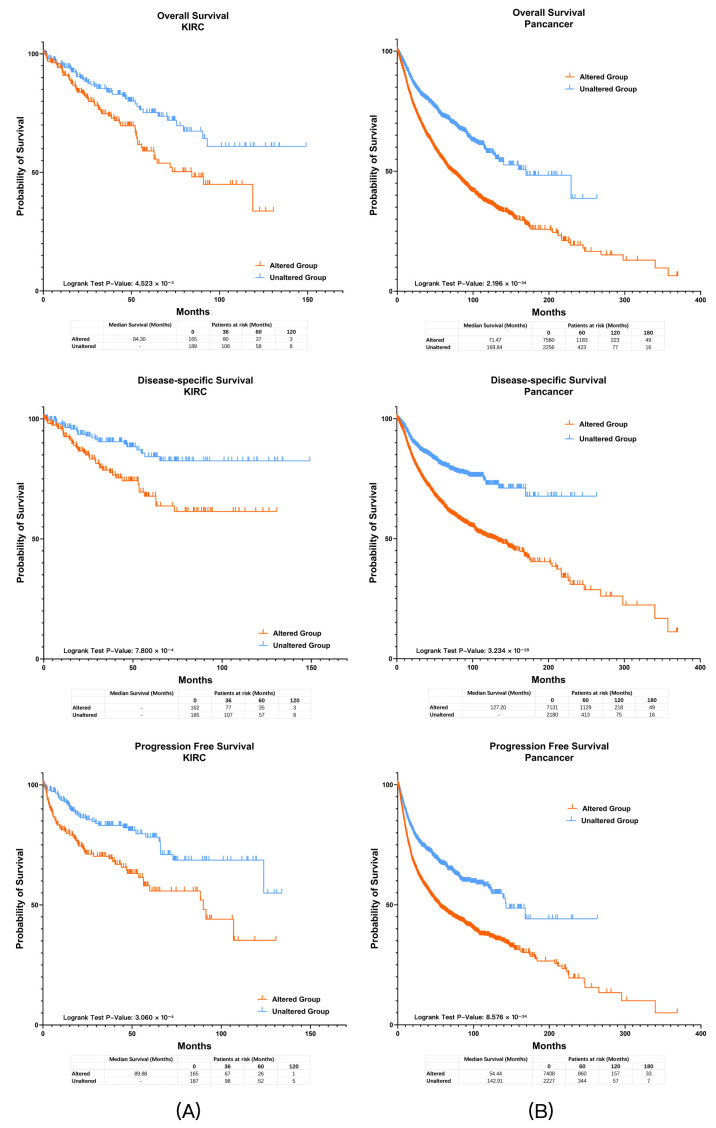
Survival analysis of Panel 3 in ccRCC and pan-cancer cohorts. (**A**) Kaplan–Meier curves showing overall survival (OS), disease-specific survival (DSS), and progression-free survival (PFS) in the TCGA-KIRC assessment cohort stratified by Panel 3 status. Patients in the altered group exhibited worse OS, DSS, and PFS compared with the unaltered group (all q < 0.05). (**B**) Prognostic significance was further confirmed in pan-cancer populations from TCGA datasets, where the altered group consistently demonstrated inferior survival outcomes (all q < 0.05).

Panel 3 incorporated 39 drug-related genes in addition to the genomic alterations included in Panel 2. In the TCGA-KIRC assessment cohort, 166 patients (46.9%) met the genetic criteria of Panel 3, and the altered group demonstrated worse OS, DSS, and PFS (Figure 4A; all q < 0.05). There were no significant differences between groups in terms of sex (*p* = 0.715), tumor grade (*p* = 0.185), or tumor stage (*p* = 0.610). The prognostic effect of Panel 3 was further supported by univariate and multivariate Cox regression analyses, and remained significant after Holm correction for multiple testing (adjusted *p* < 0.05).

To further assess incremental prognostic value beyond conventional pathological variables, time-dependent ROC analyses were performed at 1, 3, and 5 years for OS, DSS, and PFS. Compared with the pathology-based baseline model (pT stage + tumor grade), incorporation of Panels 1–3 resulted in consistent, though modest, improvements in AUC across endpoints. These findings suggest a potential complementary role of the panels in molecular risk stratification. However, the magnitude of improvement is limited and should be interpreted with caution (Supplementary Material File S1, Supplementary Figures S2–S4).

#### 3.2.2. Survival Verification in Pan-Cancer Population

We further examined the prognostic efficacy of the panels for pan-cancer patients. There were 9896 patients with complete gene mutation and CNV data in the TCGA Pan-Cancer Atlas Database, of which 9816 patients had complete OS data, 9311 patients had complete DSS data, and 9635 patients had complete PFS data. In the TCGA Pan-Cancer Atlas dataset (Figure 4B), 7616 patients (77.0%) were classified as altered by Panel 3, and they consistently demonstrated worse OS, DSS, and PFS (all q < 0.05). In the PAWG cohort (only 291 patients had complete mutation, CNV and OS data from the PAWG database), 196 patients (67.4%) were classified as altered by Panel 3, and these patients showed inferior OS compared with the unaltered group (q < 0.05).

#### 3.2.3. Multivariate Survival Analysis (Cox Regression)

To externally evaluate the prognostic relevance of the panels, univariate and multivariate Cox regression analyses were performed in the TCGA-KIRC cohort. Given that the panels were derived from patients with localized clear-cell renal cell carcinoma (ccRCC) with venous tumor thrombus and intermediate tumor grade (G2/G3), prognostic analyses were conducted across three clinically relevant subsets: localized patients without distant metastasis, patients with intermediate tumor grade (G2/G3), and the overall ccRCC population.

**Figure 5:**
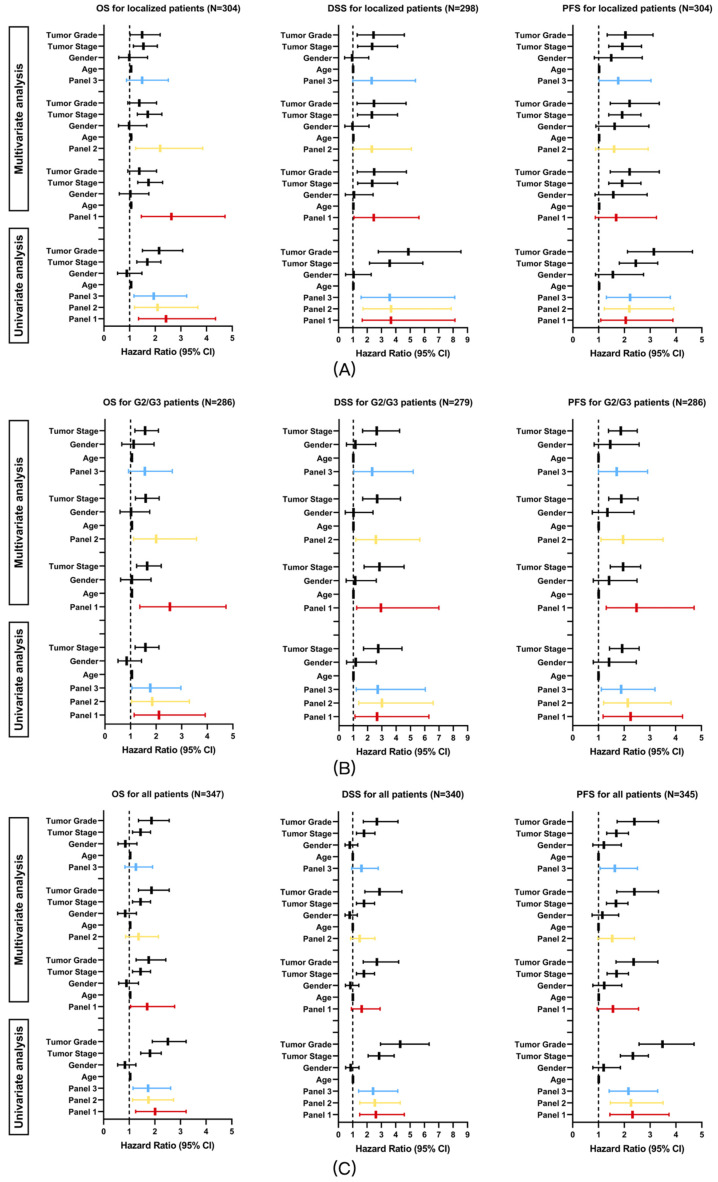
Univariate and multivariate Cox regression analyses of Panels 1–3 for OS, DSS, and PFS in the TCGA-KIRC cohort. Analyses were performed in localized patients (**A**), intermediate-grade (G2/G3) patients (**B**), and the overall ccRCC population (**C**). Hazard ratios and 95% confidence intervals were adjusted for tumor stage, tumor grade, age, and sex in multivariate models. Adjusted *p*-values are provided in Supplementary Table S9.

Univariate and multivariate Cox regression results for OS, DSS, and PFS are summarized in Figure 5A–C. In univariate analyses, all three panels were associated with OS, DSS, and PFS across all analyzed subgroups (all q < 0.05).

In multivariate Cox regression models adjusting for tumor stage, tumor grade, age, and sex, the panels retained prognostic relevance in specific clinical contexts. Among localized patients, after Holm correction for multiple testing, Panel 1 and Panel 2 remained independently associated with OS (Panel 1, adjusted *p* = 0.003; Panel 2, adjusted *p* = 0.014), whereas the associations with DSS and PFS did not remain significant (all adjusted *p* > 0.05).

In the intermediate-grade (G2/G3) subgroup, after Holm correction, all three panels remained associated with DSS and PFS (all adjusted *p* < 0.05), whereas for OS, only Panel 1 and Panel 2 remained significant (Panel 1, adjusted *p* = 0.009; Panel 2, adjusted *p* = 0.038; Panel 3, adjusted *p* = 0.097). In the overall study population, all panels showed consistent prognostic trends; however, none of the associations remained significant after Holm correction (all adjusted *p* > 0.05). Panel 3 also showed an association with PFS before correction, but this did not remain significant after Holm adjustment (adjusted *p* = 0.075) and should therefore be interpreted cautiously.

#### 3.2.4. Association with Therapy-Related Genomic Biomarkers

In clinical practice, HRD, TMB, and MSI are recognized biomarkers for predicting systemic treatment efficacy. Higher HRD predicts sensitivity to PARP inhibitors and platinum-based chemotherapy [20], while elevated TMB and MSI are associated with improved responsiveness to immune checkpoint inhibitors [21,22,23]. For TMB, MSI, and HRD analysis, all calculations were performed at the pan-cancer level. TMB-high was defined as ≥10 mutations/Mb [21]. In the TCGA Pan-Cancer dataset, 9896 samples had complete mutation, CNV, and TMB data; 1269 (12.82%) were TMB-high. In the PAWG dataset, 2670 samples had complete data; 135 (5.05%) were TMB-high. MSI-high was defined as an MSI score ≥ 10 [22,23]. In the TCGA Pan-Cancer dataset, 9340 samples had complete mutation, CNV, and MSI data; 291 (3.12%) were MSI-high. HRD-high was defined as an HRD score ≥ 42 [20]. In the TCGA Pan-Cancer dataset, 9623 samples had complete mutation, CNV, and HRD data; 1126 (11.70%) were HRD-high. All analyses were conducted using the TCGA Pan-Cancer Atlas and PAWG cohorts for consistent cross-validation.

In our analysis, patients classified as altered by Panels 1 and 2 exhibited higher HRD, TMB, and MSI scores than the unaltered groups (all q < 0.05), suggesting potential therapeutic relevance.

**Figure 6:**
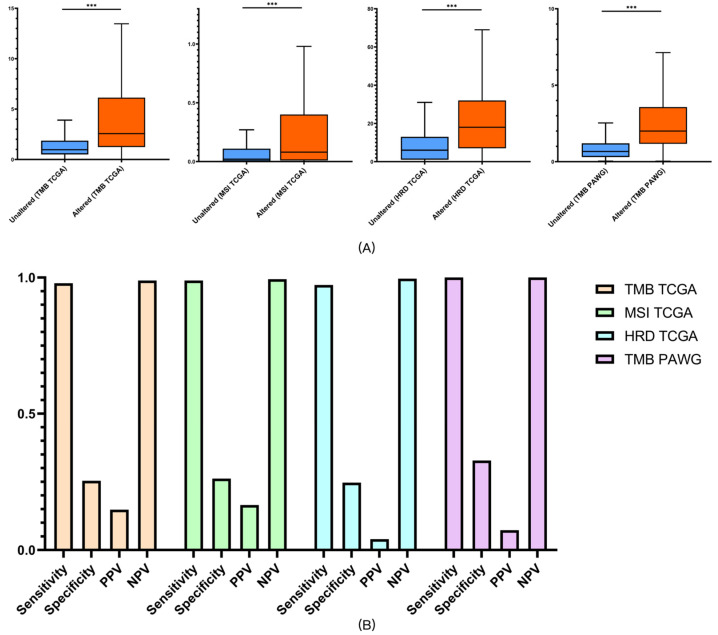
Predictive performance of Panel 3 for therapeutic biomarkers. (**A**) TMB, MSI scores, and HRD in TCGA and TMB in PAWG, stratified by Panel 3 status. (**B**) Sensitivity, specificity, PPV, and NPV of Panel 3 for predicting treatment biomarkers in TCGA and PAWG cohorts. *** *p* < 0.001.

Panel 3, which incorporated 39 drug-related genes on top of Panel 2, demonstrated the strongest predictive performance. In the TCGA cohort, altered patients showed higher HRD, TMB, and MSI scores (all q < 0.05), while in the PAWG dataset, altered patients demonstrated higher TMB compared with the unaltered group (Figure 6A; all q < 0.05). Similarly, all biomarker differences remained significant after FDR correction (all q < 0.05; Supplementary Table S8).

Sensitivity, specificity, positive predictive value (PPV), and negative predictive value (NPV) were calculated to further evaluate predictive accuracy. Panel 3 achieved sensitivities of 97.9% for HRD, 98.9% for TMB, and 97.3% for MSI in TCGA, with corresponding NPVs of 98.9%, 99.4%, and 99.6%. In the PAWG dataset, Panel 3 reached 100% sensitivity and 100% NPV for predicting high TMB (≥10/Mb). Although specificity and PPV were modest, which is to be expected due to the low prevalence of treatment response, these findings support the clinical utility of Panel 3 primarily as a rule-out tool for identifying patients unlikely to benefit from systemic treatment, thereby helping avoid ineffective therapy [24] (Figure 6B). Because predictive values vary with the prevalence of true responders, the high NPV observed here should be interpreted in this context.

## 4. Discussion

As a special subtype of renal carcinoma, patients with tumor thrombi usually experience poor prognosis [7,8]. Several studies have systematically investigated the genomic characteristics of this population; however, marked heterogeneity among studies has limited the clinical translation of their findings, and no clinically practical gene panel has yet been established [25,26,27]. In our study, to ensure a clear genetic contrast for developing an easy-to-use clinical tool, we strictly limited tumor grade (G2 or G3) and size (pT1, <7 cm), and compared whole-exome sequencing (WES) data between patients with thrombi and those with conventional ccRCC. Dual external pan-cancer assessment confirmed that the panels we constructed provided reliable prognostic value for both survival and drug efficacy. However, this analysis was intended to test robustness rather than tumor-agnostic clinical applicability.

Given that the panels were derived from ccRCC-specific biological contexts, their primary clinical relevance remains within ccRCC. Although Panel 1 identified the smallest altered population of the three panels, it showed the best survival prognostic accuracy. Building on Panels 1 and 2, Panel 3 encompassed the largest altered population and additionally provided treatment-relevant genomic information, particularly for pathways related to PARP inhibitors, platinum chemotherapy, and immunotherapy, suggesting potential applicability in informing exploratory assessments of systemic treatment–related biomarkers. However, these analyses were based exclusively on associations with established genomic surrogate biomarkers rather than on actual treatment outcomes and should therefore be interpreted as exploratory and hypothesis-generating rather than predictive of clinical therapeutic efficacy. Notably, some SMGs were from the KRT/KRTAP family. As such alterations are often regarded as biologically indolent or related to sequencing in solid tumors, they were interpreted cautiously and have not been emphasized in this study. Importantly, these genes were not considered core biological drivers of malignancy, and the construction and validation of the panels were not dependent on any single gene or gene family. Instead, panel performance was driven by the aggregated genomic signal and its reproducibility across independent external cohorts.

With the growing emphasis on precision oncology, our panels may support several clinical applications. First, rather than replacing traditional histopathologic grading, the panels are intended to provide complementary molecular information that augments conventional risk stratification, particularly in clinically ambiguous settings where morphology alone may be insufficient, and panels can be readily combined with artificial intelligence for large-scale deployment. Second, because the panels were derived from small-diameter ccRCC, they may provide additional molecular context for risk assessment in settings such as active surveillance or minimally invasive management, pending further validation. Third, Panel 3 covers therapeutic targets of tyrosine kinase inhibitors (TKIs) and mTOR inhibitors, facilitating rational drug selection. Fourth, given the observed high sensitivity and negative predictive value (NPV) for HRD, TMB, and MSI, the panels are associated with genomic features that have been linked to responsiveness to systemic therapies in prior studies, although prospective clinical validation is required. Finally, as the dichotomous panels were developed from WES data (average depth = 162×) of FFPE samples, translation to routine clinical use requires only low-depth targeted sequencing of FFPE tissue, ensuring high feasibility and markedly lower cost compared with conventional panels.

This study has limitations. First, because ccRCC patients meeting the strict inclusion criteria are relatively rare, the overall sample size was limited. Second, the analysis was retrospective and derived from a single center. Accordingly, the study was not designed to support causal or mechanistic inference at the individual gene level, and conclusions were deliberately drawn at the multigene panel level with emphasis on external reproducibility. Third, although external assessment was performed using two large pan-cancer datasets, the real-world clinical utility of these panels requires confirmation through prospective, multicenter studies. It should also be emphasized that the panels were primarily designed and validated for prognostic stratification, whereas their ability to predict treatment-related biomarkers represents an exploratory secondary analysis. These findings suggest broader translational potential but require further clinical validation before adoption for therapeutic decision-making. Additionally, although the primary tumor and thrombus samples were paired, the limited cohort size precluded the use of formal paired statistical models. More stringent significance thresholds (*p* < 0.0025, q < 0.04) were therefore applied to minimize false positives, which partially compensates for non-independence but should be validated in larger cohorts. The gene-level harmonization strategy may introduce potential classification bias by simplifying regional CNV events into single representative genes. Minor classification bias from CNV harmonization may also persist and warrants optimization in future studies. Furthermore, as PPV and NPV depend on the prevalence of therapy-responsive cases, the high NPV mainly reflects the low prevalence of high HRD, TMB, or MSI status in the analyzed cohorts and should be interpreted cautiously. Accordingly, the current analyses should be regarded as exploratory, and prospective multicenter studies integrating molecular panels with established pathological models will be required before clinical implementation.

## 5. Conclusions

In conclusion, our study characterized the distinctive genomic landscape of small-diameter ccRCC with venous tumor thrombus and established three translational panels derived from real-world sequencing data. Through dual external assessment, these panels demonstrated robust prognostic value for survival outcomes across ccRCC and pan-cancer populations, supporting their complementary clinical relevance in molecular risk stratification. Of the three panels, Panel 3 showed the greatest practical applicability and also provided exploratory evidence for predicting therapeutic biomarkers such as HRD, TMB, and MSI, suggesting potential use in guiding systemic treatment decisions. Importantly, these pathology-driven molecular panels are compatible with routine FFPE tissue sampling, offering a low-cost, high-accuracy, and clinically feasible solution. By integrating morphological assessment with genomic profiling, our work exemplifies the principles of modern precision pathology and provides a practical framework for companion diagnostics in personalized oncology.

## 6. Patents

Xiong G., He S., He Z., Zhou L. A gene panel for grading human tumors and its applications. Chinese Patent: ZL 2021 1 0762353.5. Filed 6 July 2021, granted 22 November 2022. Assignee: Peking University First Hospital (CN 113,355,422 B) [28].

Xiong G., He S., He Z., Zhou L. Gene combinations for classification of human tumors and their use. Japanese Patent: JP 7,667,865 B2. Application No. 2023-544465, filed 2 March 2022, granted 15 April 2025. Assignee: Peking University First Hospital [29].

Both patents are directly related to the gene panels developed in this study, supporting their translational potential in modern precision pathology. Patents related to the panels have been disclosed and did not influence the study design, data analysis, interpretation, or reporting, and no commercial entity was involved in this work.

## Data Availability

The original sequencing data (Bam files) have been deposited at the National Genomics Data Center, Beijing Institute of Genomics (BIG), Chinese Academy of Sciences “https://ngdc.cncb.ac.cn/”, under accession number PRJCA010456 “https://ngdc.cncb.ac.cn/gsa-human/browse/HRA003005 (accessed on 10 September 2022)”. Access may be granted upon request submitted through the website’s standard process, which will be considered by the Scientific Research Office of Peking University First Hospital for data sharing.

## Supplementary Materials

The following supporting information can be downloaded at: https://www.mdpi.com/article/10.3390/diagnostics16070989/s1, Supplementary Material File S1: Supplementary Table S1: Sequencing information of all patients. Table S2: Architecture of Panel 1. Table S3: Architecture of Panel 2. Table S4: Architecture of Panel 3. Table S5: SMGs of primary tumors (The high-risk group, FDR CT < 0.2). Table S6: SMGs of tumor thrombi (The High-risk group, FDR CT < 0.2). Table S7: SMGs of the control group (FDR CT < 0.2). Table S8: FDR-adjusted *p*-values for survival validation and biomarker analyses. Table S9: Holm-adjusted *p*-values for panel variables in multivariate Cox models. Figure S1: Drug Targets Analysis. Figure S2: Time-dependent ROC curves (OS). Figure S3: Time-dependent ROC curves (DSS). Figure S4: Time-dependent ROC curves (PFS). Supplementary Data S1. Drug Susceptibility.xlsx; Supplementary Data S2. CNV Analysis.xlsx; Supplementary Data S3. Somatic Signatures Analysis.xlsx; Supplementary Data S4. KEGG Pathway Enrichment Analysis.xlsx. References [30,31,32,33,34] are cited in the supplementary materials.

## Abbreviations

The following abbreviations are used in this manuscript:

ccRCC	clear-cell renal cell carcinoma
ccRCC-TT	clear-cell renal cell carcinoma with tumor thrombus
CNV	copy number variation
CR	complete response
DSS	disease-specific survival
FDR	false discovery rate
FFPE	formalin-fixed paraffin-embedded
HRD	homologous recombination deficiency
MSI	microsatellite instability
NPV	negative predictive value
OS	overall survival
PARP	poly(ADP-ribose) polymerase
PAWG	Pan-cancer Analysis of Whole Genomes
PFS	progression-free survival
PPV	positive predictive value
TCGA	The Cancer Genome Atlas
TMB	tumor mutational burden
TKI	tyrosine kinase inhibitor
WES	whole-exome sequencing
SMG	significantly mutated gene

## References

[1] Siegel R.L., Kratzer T.B., Giaquinto A.N., Sung H., Jemal A. (2025). Cancer statistics, 2025. CA Cancer J. Clin..

[2] Beck S.D., Patel M.I., Snyder M.E., Kattan M.W., Motzer R.J., Reuter V.E., Russo P. (2004). Effect of papillary and chromophobe cell type on disease-free survival after nephrectomy for renal cell carcinoma. Ann. Surg. Oncol..

[3] Capitanio U., Cloutier V., Zini L., Isbarn H., Jeldres C., Shariat S.F., Perrotte P., Antebi E., Patard J.J., Montorsi F. (2009). A critical assessment of the prognostic value of clear cell, papillary and chromophobe histological subtypes in renal cell carcinoma: A population-based study. BJU Int..

[4] Keegan K.A., Schupp C.W., Chamie K., Hellenthal N.J., Evans C.P., Koppie T.M. (2012). Histopathology of surgically treated renal cell carcinoma: Survival differences by subtype and stage. J. Urol..

[5] Fuhrman S.A., Lasky L.C., Limas C. (1982). Prognostic significance of morphologic parameters in renal cell carcinoma. Am. J. Surg. Pathol..

[6] Lang H., Lindner V., de Fromont M., Molinie V., Letourneux H., Meyer N., Martin M., Jacqmin D. (2005). Multicenter determination of optimal interobserver agreement using the Fuhrman grading system for renal cell carcinoma: Assessment of 241 patients with >15-year follow-up. Cancer.

[7] Chen Z., Yang F., Ge L., Qiu M., Liu Z., Liu C., Tian X., Zhang S., Ma L. (2021). Outcomes of renal cell carcinoma with associated venous tumor thrombus: Experience from a large cohort and short time span in a single center. BMC Cancer.

[8] Tang Q., Song Y., Li X., Meng M., Zhang Q., Wang J., He Z., Zhou L. (2015). Prognostic Outcomes and Risk Factors for Patients with Renal Cell Carcinoma and Venous Tumor Thrombus after Radical Nephrectomy and Thrombectomy: The Prognostic Significance of Venous Tumor Thrombus Level. Biomed. Res. Int..

[9] Sobin L.H., Gospodarowicz M.K., Wittekind C., International Union Against Cancer (2009). TNM Classification of Malignant Tumours.

[10] Uzosike A.C., Patel H.D., Alam R., Schwen Z.R., Gupta M., Gorin M.A., Johnson M.H., Gausepohl H., Riffon M.F., Trock B.J. (2018). Growth Kinetics of Small Renal Masses on Active Surveillance: Variability and Results from the DISSRM Registry. J. Urol..

[11] Liu J., Lichtenberg T., Hoadley K.A., Poisson L.M., Lazar A.J., Cherniack A.D., Kovatich A.J., Benz C.C., Levine D.A., Lee A.V. (2018). An Integrated TCGA Pan-Cancer Clinical Data Resource to Drive High-Quality Survival Outcome Analytics. Cell.

[12] The ICGC/TCGA Pan-Cancer Analysis of Whole Genomes Consortium (2020). Pan-cancer analysis of whole genomes. Nature.

[13] Wang A., Wu L., Lin J., Han L., Bian J., Wu Y., Robson S.C., Xue L., Ge Y., Sang X. (2018). Whole-exome sequencing reveals the origin and evolution of hepato-cholangiocarcinoma. Nat. Commun..

[14] Escudier B., Eisen T., Stadler W.M., Szczylik C., Oudard S., Siebels M., Negrier S., Chevreau C., Solska E., Desai A.A. (2007). Sorafenib in advanced clear-cell renal-cell carcinoma. N. Engl. J. Med..

[15] Motzer R.J., Hutson T.E., Tomczak P., Michaelson M.D., Bukowski R.M., Rixe O., Oudard S., Negrier S., Szczylik C., Kim S.T. (2007). Sunitinib versus interferon alfa in metastatic renal-cell carcinoma. N. Engl. J. Med..

[16] Hudes G., Carducci M., Tomczak P., Dutcher J., Figlin R., Kapoor A., Staroslawska E., Sosman J., McDermott D., Bodrogi I. (2007). Temsirolimus, interferon alfa, or both for advanced renal-cell carcinoma. N. Engl. J. Med..

[17] Cerami E., Gao J., Dogrusoz U., Gross B.E., Sumer S.O., Aksoy B.A., Jacobsen A., Byrne C.J., Heuer M.L., Larsson E. (2012). The cBio cancer genomics portal: An open platform for exploring multidimensional cancer genomics data. Cancer Discov..

[18] Gao J., Aksoy B.A., Dogrusoz U., Dresdner G., Gross B., Sumer S.O., Sun Y., Jacobsen A., Sinha R., Larsson E. (2013). Integrative analysis of complex cancer genomics and clinical profiles using the cBioPortal. Sci. Signal.

[19] Zigeuner R., Hutterer G., Chromecki T., Imamovic A., Kampel-Kettner K., Rehak P., Langner C., Pummer K. (2010). External validation of the Mayo Clinic stage, size, grade, and necrosis (SSIGN) score for clear-cell renal cell carcinoma in a single European centre applying routine pathology. Eur. Urol..

[20] Gonzalez-Martin A., Pothuri B., Vergote I., DePont Christensen R., Graybill W., Mirza M.R., McCormick C., Lorusso D., Hoskins P., Freyer G. (2019). Niraparib in Patients with Newly Diagnosed Advanced Ovarian Cancer. N. Engl. J. Med..

[21] Subbiah V., Solit D.B., Chan T.A., Kurzrock R. (2020). The FDA approval of pembrolizumab for adult and pediatric patients with tumor mutational burden (TMB) >/=10: A decision centered on empowering patients and their physicians. Ann. Oncol..

[22] Abida W., Cheng M.L., Armenia J., Middha S., Autio K.A., Vargas H.A., Rathkopf D., Morris M.J., Danila D.C., Slovin S.F. (2019). Analysis of the Prevalence of Microsatellite Instability in Prostate Cancer and Response to Immune Checkpoint Blockade. JAMA Oncol..

[23] Latham A., Srinivasan P., Kemel Y., Shia J., Bandlamudi C., Mandelker D., Middha S., Hechtman J., Zehir A., Dubard-Gault M. (2019). Microsatellite Instability Is Associated With the Presence of Lynch Syndrome Pan-Cancer. J. Clin. Oncol..

[24] Goldman M.J., Craft B., Hastie M., Repecka K., McDade F., Kamath A., Banerjee A., Luo Y., Rogers D., Brooks A.N. (2020). Visualizing and interpreting cancer genomics data via the Xena platform. Nat. Biotechnol..

[25] Wang X.M., Lu Y., Song Y.M., Dong J., Li R.Y., Wang G.L., Wang X., Zhang S.D., Dong Z.H., Lu M. (2020). Integrative genomic study of Chinese clear cell renal cell carcinoma reveals features associated with thrombus. Nat. Commun..

[26] Warsow G., Hubschmann D., Kleinheinz K., Nientiedt C., Heller M., Van Coile L., Tolstov Y., Trennheuser L., Wieczorek K., Pecqueux C. (2018). Genomic features of renal cell carcinoma with venous tumor thrombus. Sci. Rep..

[27] Niu S., Liu K., Xu Y., Peng C., Yu Y., Huang Q., Wu S., Cui B., Huang Y., Ma X. (2021). Genomic Landscape of Chinese Clear Cell Renal Cell Carcinoma Patients with Venous Tumor Thrombus Identifies Chromosome 9 and 14 Deletions and Related Immunosuppressive Microenvironment. Front. Oncol..

[28] Xiong G., He S., He Z., Zhou L. (2022). A Gene Panel for Grading Human Tumors and Its Applications. Chinese Patent.

[29] Xiong G., He S., He Z., Zhou L. (2025). Gene Combinations for Classification of Human Tumors and Their Use. Japanese Patent.

[30] Forbes S.A., Beare D., Boutselakis H., Bamford S., Bindal N., Tate J., Cole C.G., Ward S., Dawson E., Ponting L. (2017). COSMIC: Somatic cancer genetics at high-resolution. Nucleic Acids Res..

[31] Chakravarty D., Gao J., Phillips S.M., Kundra R., Zhang H., Wang J., Rudolph J.E., Yaeger R., Soumerai T., Nissan M.H. (2017). OncoKB: A Precision Oncology Knowledge Base. JCO Precis. Oncol..

[32] Sondka Z., Bamford S., Cole C.G., Ward S.A., Dunham I., Forbes S.A. (2018). The COSMIC Cancer Gene Census: Describing genetic dysfunction across all human cancers. Nat. Rev. Cancer.

[33] Rahman N. (2014). Realizing the promise of cancer predisposition genes. Nature.

[34] Huang K.L., Mashl R.J., Wu Y., Ritter D.I., Wang J., Oh C., Paczkowska M., Reynolds S., Wyczalkowski M.A., Oak N. (2018). Pathogenic germline variants in 10,389 adult cancers. Cell.

