# Supplementary material for: Pathology-Driven Genomic Panels for Personalized Prognostic Stratification and Exploratory Therapeutic Prediction in Clear-Cell Renal Cell Carcinoma with Tumor Thrombus
Source: Diagnostics (Basel). 2026 Mar 25;16(7):989. doi: 10.3390/diagnostics16070989 (PMC13073698; doi:10.3390/diagnostics16070989)
Supplement: Supplementary file 1 [file diagnostics-16-00989-s001.zip › diagnostics-4076717-supplementary file S1.pdf]

Supplementary Materials for

Pathology-Driven Genomic Panels for Personalized Prognostic Stratification and Exploratory Therapeutic Prediction in Clear Cell Renal Cell Carcinoma with Tumor Thrombus

Chenghao Tan *et al.*

*Corresponding author:
Shiming He, shiminghe@bjmu.edu.cn
Gengyan Xiong, xgy6205@gmail.com;

This file includes:

Contents
Supplementary Tables S1-S9
Supplementary Figure S1-S4

Other Supplementary Materials for this manuscript include the following:

Separate data files (Supplementary Data S1-S4):
Supplementary Data S1 Drug Susceptibility.xlsx
Supplementary Data S2 CNV Analysis.xlsx
Supplementary Data S3 Somatic Signatures Analysis.xlsx
Supplementary Data S4 KEGG Pathway Enrichment Analysis.xlsx

Contents

Supplementary Table S1 Sequencing information of all patients
………………………………………………………………………………………...................... 3
Supplementary Table S2 Architecture of Panel 1
………………………………………………………………………………………...................... 4
Supplementary Table S3 Architecture of Panel 2
………………………………………………………………………………………...................... 5
Supplementary Table S4 Architecture of Panel 3
………………………………………………………………………………………...................... 6
Supplementary Table S5 SMGs of primary tumors (The high-risk group, FDR CT < 0.2)
………………………………………………………………………………………...................... 7
Supplementary Table S6 SMGs of tumor thrombi (The High-risk group, FDR CT < 0.2)
………………………………………………………………………………………...................... 8
Supplementary Table S7 SMGs of the control group (FDR CT < 0.2)
………………………………………………………………………………………...................... 9
Supplementary Table S8 FDR-adjusted p-values for survival validation and biomarker analyses
………………………………………………………………………………………...................... 10
Supplementary Table S9 Holm-adjusted p-values for panel variables in multivariate Cox models
………………………………………………………………………………………...................... 11
Supplementary Figure S1 Drug Targets Analysis
………………………………………………………………………………………...................... 12
Supplementary Figure S2 Time-dependent ROC curves (OS)
………………………………………………………………………………………...................... 13
Supplementary Figure S3 Time-dependent ROC curves (DSS)
………………………………………………………………………………………...................... 14
Supplementary Figure S4 Time-dependent ROC curves (PFS)
………………………………………………………………………………………...................... 15
Definition and Selection of SMGs, Driver Genes, and Predisposing Genes
………………………………………………………………………………………...................... 16

Supplementary Table S1 Sequencing information of all patients

Patient	Sample	Sample type	Sequencing depth (×)	Patient	Sample	Sample type	Sequencing depth (×)
P1	RH1	Primary tumor	129	P11	RH31	Adjacent normal tissue	144
	RH2	Adjacent normal tissue	148		RH32	Primary tumor	144
	RH3	Tumor thrombus	148				
P2	RH4	Tumor thrombus	178	P12	RH33	Adjacent normal tissue	163
	RH5	Primary tumor	114		RH34	Primary tumor	150
	RH6	Adjacent normal tissue	142				
P3	RH7	Tumor thrombus	170	P13	RH35	Adjacent normal tissue	159
	RH8	Primary tumor	150		RH36	Primary tumor	127
	RH9	Adjacent normal tissue	157				
P4	RH10	Tumor thrombus	182	P14	RH37	Adjacent normal tissue	171
	RH11	Primary tumor	312		RH38	Primary tumor	138
	RH12	Adjacent normal tissue	175				
P5	RH13	Primary tumor	175	P15	RH39	Adjacent normal tissue	154
	RH14	Tumor thrombus	162		RH40	Primary tumor	178
	RH15	Adjacent normal tissue	174				
P6	RH16	Primary tumor	162	P16	RH41	Adjacent normal tissue	145
	RH17	Adjacent normal tissue	159		RH42	Primary tumor	197
	RH18	Tumor thrombus	163				
P7	RH19	Primary tumor	170	P17	RH43	Adjacent normal tissue	148
	RH20	Adjacent normal tissue	161		RH44	Primary tumor	145
	RH21	Tumor thrombus	187				
P8	RH22	Primary tumor	132	P18	RH45	Adjacent normal tissue	154
	RH23	Adjacent normal tissue	152		RH46	Primary tumor	161
	RH24	Tumor thrombus	216				
P9	RH25	Primary tumor	171	P19	RH47	Adjacent normal tissue	173
	RH26	Tumor thrombus	139		RH48	Primary tumor	158
	RH27	Adjacent normal tissue	291				
P10	RH28	Primary tumor	109	P22	RH53	Adjacent normal tissue	166
	RH29	Tumor thrombus	110		RH54	Primary tumor	165
	RH30	Adjacent normal tissue	125				

Supplementary Table S2 Architecture of Panel 1

Key genes	DNA amplification segments		
	Cytoband	Wide Peak Boundaries	The gene (DNA amplification) verified in the TCGA Pan-cancer database
MAF	11p15.1	chr11:17739501-17760249	MYOD1
WBP2NL	12p13.31	chr12:7269501-7310249	CLSTN3
ZNF20	12p13.33	chr12:169501-250249	IQSEC3
KRT9	12q13.13	chr12:54329501-54350249	HOXC13
TRIM26	13q34	chr13:114519501-	GAS6
SCYL1	15q24.1	chr15:74209501-74220249	LOXL1
SLC16A6	15q24.1	chr15:73649501-73670249	HCN4
SRC	15q25.1	chr15:78409501-78430249	CIB2
EGR1	15q25.2	chr15:83859501-83880249	HDGFL3 (HDGFRP3)
KNOP1	18p11.22	chr18:8809501-8820249	MTCL1 (CCDC165)
MNS1	20q13.33	chr20:62189501-62200249	HELZ2 (PRIC285)
KRTAP10-10	21q22.3	chr21:45949501-46110249	TSPEAR
FAM71E2	22q11.21	chr22:19499501-19760249	SEPTIN5 (SEPT5)
PROSER3	22q13.1	chr22:36649501-38700249	MYH9
TMEM82	22q13.31	chr22:46309501-46330249	WNT7B
CCDC136	2p25.3	chr2:3679501-3700249	COLEC11
KRT76	2q31.2	chr2:179479501-179610249	TTN
IFITM3	2q35	chr2:219719501-219840249	WNT6
GLIS1	3q21.3	chr3:126249501-126270249	CHST13
U2AF2	3q22.1	chr3:129319501-129330249	PLXND1
USP35	3q23	chr3:138659501-138770249	FOXL2
ZNF700	3q27.1	chr3:183999501-184020249	PSMD2
TCEAL5	4p16.1	chr4:8579501-8590249	GPR78
WDR44	4p16.1	chr4:9319501-9330249	USP17L5
CALML6	4p16.3	chr4:1189501-1230249	CTBP1
	5q33.1	chr5:150899501-150940249	FAT2
	6p22.2	chr6:26519501-26530249	HCG11
	6q13	chr6:71659501-71670249	B3GAT2
	6q13	chr6:73329501-73340249	KCNQ5
	6q24.3	chr6:147819501-147840249	SAMD5
	6q25.3	chr6:157089501-157110249	ARID1B
	6q27	chr6:164889501-164900249	C6orf118
	7q22.1	chr7:100539501-100560249	ACHE
	8p12	chr8:29189501-29200249	DUSP4
	8p21.3	chr8:21999501-22070249	BMP1
	8p23.3	chr8:1939501-1960249	KBTBD11
	9q22.1	chr9:91789501-91800249	SHC3
	10q24.2	chr10:99419501-99440249	PI4K2A
	11q12.3	chr11:63329501-63350249	PLAAT2 (HRASLS2)
	19p12	chr19:24059501-24070249	ZNF726
	19p13.3	chr19:4229501-4250249	EBI3
	19q13.32	chr19:46879501-46900249	PPP5C
	20p11.21	chr20:22559501-22570249	FOXA2
	2q33.3	chr2:207989501-208000249	KLF7
	6p22.3	chr6:20399501-20410249	E2F3

Supplementary Table S3 Architecture of Panel 2

Key genes	DNA amplification segments		
	Cytoband	Wide Peak Boundaries	The gene (DNA amplification) verified in the TCGA Pan-cancer database
ASAH1	11p15.1	chr11:17739501-17760249	MYOD1
CALML6	12p13.31	chr12:7269501-7310249	CLSTN3
CCDC136	12p13.33	chr12:169501-250249	IQSEC3
CIDEC	12q13.13	chr12:54329501-54350249	HOXC13
COX18	13q34	chr13:114519501-	GAS6
EGR1	15q24.1	chr15:74209501-74220249	LOXL1
FAM71E2	15q24.1	chr15:73649501-73670249	HCN4
FKBP7	15q25.1	chr15:78409501-78430249	CIB2
GLIS1	15q25.2	chr15:83859501-83880249	HDGFL3 (HDGFRP3)
IFITM3	18p11.22	chr18:8809501-8820249	MTCL1 (CCDC165)
IMMT	20q13.33	chr20:62189501-62200249	HELZ2 (PRIC285)
KNOP1	21q22.3	chr21:45949501-46110249	TSPEAR
KRT76	22q11.21	chr22:19499501-19760249	SEPTIN5 (SEPT5)
KRT9	22q13.1	chr22:36649501-38700249	MYH9
KRTAP10-	22q13.31	chr22:46309501-46330249	WNT7B
KRTAP10-8	2p25.3	chr2:3679501-3700249	COLEC11
MAF	2q31.2	chr2:179479501-	TTN
MFRP	2q35	chr2:219719501-	WNT6
MNS1	3q21.3	chr3:126249501-	CHST13
PRB2	3q22.1	chr3:129319501-	PLXND1
PROSER3	3q23	chr3:138659501-	FOXL2
REXO1	3q27.1	chr3:183999501-	PSMD2
SCYL1	4p16.1	chr4:8579501-8590249	GPR78
SLC16A6	4p16.1	chr4:9319501-9330249	USP17L5
SRC	4p16.3	chr4:1189501-1230249	CTBP1
TCEAL5	5q33.1	chr5:150899501-	FAT2
TMEM82	6p22.2	chr6:26519501-26530249	HCG11
TRIM26	6q13	chr6:71659501-71670249	B3GAT2
U2AF2	6q13	chr6:73329501-73340249	KCNQ5
USP35	6q24.3	chr6:147819501-	SAMD5
WBP2NL	6q25.3	chr6:157089501-	ARID1B
WDR44	6q27	chr6:164889501-	C6orf118
ZNF20	7q22.1	chr7:100539501-	ACHE
ZNF700	8p12	chr8:29189501-29200249	DUSP4
	8p21.3	chr8:21999501-22070249	BMP1
	8p23.3	chr8:1939501-1960249	KBTBD11
	9q22.1	chr9:91789501-91800249	SHC3
	10q24.2	chr10:99419501-99440249	PI4K2A
	11q12.3	chr11:63329501-63350249	PLAAT2 (HRASLS2)
	19p12	chr19:24059501-24070249	ZNF726
	19p13.3	chr19:4229501-4250249	EBI3
	19q13.32	chr19:46879501-46900249	PPP5C
	20p11.21	chr20:22559501-22570249	FOXA2
	2q33.3	chr2:207989501-	KLF7
	6p22.3	chr6:20399501-20410249	E2F3
	12q14.2	chr12:63179501-63550249	AVPR1A

Supplementary Table S4 Architecture of Panel 3

Key genes	DNA amplification segments			Targeted drugs related key genes
	Cytoband	Wide Peak Boundaries	The gene (DNA amplification) verified in the TCGA Pan-cancer database	
ASAH1	11p15.1	chr11:17739501-17760249	MYOD1	MYH11
CALML6	12p13.31	chr12:7269501-7310249	CLSTN3	NUP214
CCDC136	12p13.33	chr12:169501-250249	IQSEC3	DNMT3A
CIDEC	12q13.13	chr12:54329501-54350249	HOXC13	IDH2
COX18	13q34	chr13:114519501-114530249	GAS6	KIT
EGR1	15q24.1	chr15:74209501-74220249	LOXL1	KMT2A
FAM71E2	15q24.1	chr15:73649501-73670249	HCN4	MLLT3
FKBP7	15q25.1	chr15:78409501-78430249	CIB2	PML
GLIS1	15q25.2	chr15:83859501-83880249	HDGFL3 (HDGFRP3)	RARA
IFITM3	18p11.22	chr18:8809501-8820249	MTCL1 (CCDC165)	RBM15
IMMT	20q13.33	chr20:62189501-62200249	HELZ2 (PRIC285)	RUNX1T1
KNOP1	21q22.3	chr21:45949501-46110249	TSPEAR	RPN1
KRT76	22q11.21	chr22:19499501-19760249	SEPTIN5 (SEPT5)	MECOM
KRT9	22q13.1	chr22:36649501-38700249	MYH9	DEK
KRTAP10-10	22q13.31	chr22:46309501-46330249	WNT7B	FLT3
KRTAP10-8	2p25.3	chr2:3679501-3700249	COLEC11	MRTFA
MAF	2q31.2	chr2:179479501-179610249	TTN	KDR
MFRP	2q35	chr2:219719501-219840249	WNT6	FLT4
MNS1	3q21.3	chr3:126249501-126270249	CHST13	UGT1A1
PRB2	3q22.1	chr3:129319501-129330249	PLXND1	PDGFRB
PROSER3	3q23	chr3:138659501-138770249	FOXL2	FLT1
REXO1	3q27.1	chr3:183999501-184020249	PSMD2	CSF1R
SCYL1	4p16.1	chr4:8579501-8590249	GPR78	CYP3A5
SLC16A6	4p16.1	chr4:9319501-9330249	USP17L5	VEGFA
SRC	4p16.3	chr4:1189501-1230249	CTBP1	ASXL1
TCEAL5	5q33.1	chr5:150899501-150940249	FAT2	BCOR
TMEM82	6p22.2	chr6:26519501-26530249	HCG11	NF1
TRIM26	6q13	chr6:71659501-71670249	B3GAT2	STAG2
U2AF2	6q13	chr6:73329501-73340249	KCNQ5	TP53
USP35	6q24.3	chr6:147819501-147840249	SAMD5	U2AF1
WBP2NL	6q25.3	chr6:157089501-157110249	ARID1B	ZRSR2
WDR44	6q27	chr6:164889501-164900249	C6orf118	TET2
ZNF20	7q22.1	chr7:100539501-100560249	ACHE	RET
ZNF700	8p12	chr8:29189501-29200249	DUSP4	FGFR1
	8p21.3	chr8:21999501-22070249	BMP1	RAF1
	8p23.3	chr8:1939501-1960249	KBTBD11	BRAF
	9q22.1	chr9:91789501-91800249	SHC3	PDGFRA
	10q24.2	chr10:99419501-99440249	PI4K2A	MTOR
	11q12.3	chr11:63329501-63350249	PLAAT2 (HRASLS2)	GNAQ
	19p12	chr19:24059501-24070249	ZNF726	
	19p13.3	chr19:4229501-4250249	EBI3	
	19q13.32	chr19:46879501-46900249	PPP5C	
	20p11.21	chr20:22559501-22570249	FOXA2	
	2q33.3	chr2:207989501-208000249	KLF7	
	6p22.3	chr6:20399501-20410249	E2F3	
	12q14.2	chr12:63179501-63550249	AVPR1A	

Supplementary Table S5 SMGs of primary tumors (The high-risk group, FDR CT < 0.2)

Gene	Indels	SNVs	Tot Muts	Sample Affect	Sample Percent (%)	P-value CT	FDR CT
VHL	5	3	8	8	80	2.61E-15	5.01E-11
FLG2	8	3	11	2	20	3.29E-10	3.16E-06
KRTAP5-7	3	1	4	4	40	4.33E-07	0.002
KRT75	0	4	4	3	30	1.04E-06	0.004
KRTAP10-10	0	3	3	1	10	2.03E-06	0.006
KRTAP6-3	2	1	3	2	20	4.25E-06	0.012
KRTAP10-3	0	3	3	1	10	8.14E-06	0.017
FAM71E2	1	3	4	2	20	7.81E-06	0.017
ANKRD33	0	3	3	2	20	2.45E-05	0.047
CRIPAK	2	2	4	1	10	2.80E-05	0.049
PROSER3	0	3	3	2	20	5.95E-05	0.095
TMEM82	1	2	3	3	30	6.66E-05	0.098
CCDC136	1	4	5	3	30	7.25E-05	0.099
SERF2	0	2	2	1	10	8.60E-05	0.099
KRTAP12-3	0	2	2	2	20	8.80E-05	0.099
CSN2	2	1	3	2	20	8.82E-05	0.099
KRT76	4	0	4	4	40	9.46E-05	0.101
IFITM3	0	2	2	2	20	0.000122	0.108
PIGQ	1	2	3	2	20	0.000108	0.108
GLIS1	0	3	3	2	20	0.000124	0.108
U2AF2	1	3	4	3	30	0.000118	0.108
USP35	0	3	3	2	20	0.000145	0.12
ZNF700	3	1	4	3	30	0.000188	0.144
TCEAL5	1	1	2	2	20	0.000185	0.144
EPPK1	0	4	4	1	10	0.000199	0.147
WDR44	1	3	4	3	30	0.000225	0.16
PARP10	3	2	5	4	40	0.00024	0.164
CALML6	0	2	2	2	20	0.00025	0.165
LOC100129520	1	2	3	3	30	0.000263	0.168

Supplementary Table S6 SMGs of tumor thrombi (The High-risk group, FDR CT < 0.2)

Gene	Indels	SNVs	Tot Muts	Sample Affect	Sample Percent (%)	P-value CT	FDR CT
VHL	3	3	6	6	60	4.60E-10	4.66E-06
KRTAP5-8	5	1	6	3	30	1.18E-08	7.99E-05
PRG4	8	1	9	4	40	6.54E-08	3.32E-04
RPTN	7	0	7	2	20	1.01E-06	0.004
SETD1A	7	2	9	5	50	3.50E-06	0.011
AHNAK2	11	5	16	5	50	3.91E-06	0.011
KRTAP5-11	3	1	4	3	30	6.63E-06	0.017
MAF	0	3	3	3	30	9.06E-06	0.02
KRTAP6-1	3	0	3	3	30	1.27E-05	0.021
FOXB2	3	2	5	4	40	1.10E-05	0.021
FLG2	9	0	9	5	50	1.50E-05	0.023
WBP2NL	2	2	4	4	40	1.65E-05	0.024
TCHH	6	2	8	3	30	2.56E-05	0.035
COL11A2	3	4	7	5	50	4.92E-05	0.059
ZNF20	0	3	3	2	20	4.89E-05	0.059
HRNR	9	0	9	4	40	5.77E-05	0.063
NEFH	6	0	6	4	40	5.87E-05	0.063
KRT9	4	1	5	4	40	9.49E-05	0.087
KRTAP10-11	3	1	4	2	20	9.36E-05	0.087
FLG	7	4	11	4	40	9.04E-05	0.087
TRIM26	2	3	5	4	40	0.000101	0.089
SCYL1	5	1	6	2	20	0.000154	0.116
FAM47C	5	1	6	4	40	0.000146	0.116
ATN1	2	4	6	4	40	0.000151	0.116
AHNAK	8	5	13	5	50	0.00015	0.116
ZNF716	3	1	4	3	30	0.000171	0.124
SLC16A6	0	2	2	2	20	0.000224	0.142
SRC	0	3	3	2	20	0.000217	0.142
EGR1	4	1	5	2	20	0.000221	0.142
INO80B	3	1	4	3	30	0.000224	0.142
KNOP1	4	0	4	2	20	0.000282	0.173
MNS1	0	3	3	1	10	0.000302	0.18
PRRC2C	4	3	7	5	50	0.000328	0.19

Supplementary Table S7 SMGs of the control group (FDR CT < 0.2)

Gene	Indels	SNVs	Tot Muts	Sample Affect	Sample Percent (%)	P-value CT	FDR CT
AR	0	6	6	4	40	2.14E-12	4.10E-08
KRTAP5-3	7	0	7	3	30	6.14E-12	5.88E-08
VHL	8	1	9	9	90	3.89E-11	2.48E-07
FLG2	10	0	10	4	40	1.31E-08	6.29E-05
FLG	9	2	11	5	50	9.17E-08	2.51E-04
AHNAK2	7	5	12	7	70	4.56E-07	0.001
KRTAP5-7	2	2	4	2	20	5.57E-07	0.001
MEOX1	2	2	4	1	10	1.03E-06	0.002
RBM14-RBM4	2	1	3	3	30	5.69E-06	0.01
BAP1	2	2	4	4	40	1.81E-05	0.029
AHNAK	10	0	10	6	60	4.52E-05	0.067
KRT10	4	0	4	2	20	4.94E-05	0.068
LOC554223	0	2	2	1	10	5.53E-05	0.071
KRTAP9-1	3	0	3	3	30	9.20E-05	0.11
EVPL	6	0	6	3	30	0.000105	0.111
ZNF649	1	2	3	3	30	0.000103	0.111
COL25A1	2	2	4	3	30	0.00012	0.121
HIST1H1C	0	2	2	2	20	0.000132	0.127
KRTAP20-2	2	0	2	2	20	0.000151	0.138
TYMSOS	0	1	1	1	10	0.000189	0.158
KRTAP21-1	2	0	2	2	20	0.000201	0.16
COL4A2	2	3	5	4	40	0.000209	0.16
SERF2	0	2	2	2	20	0.000219	0.162
HRNR	4	2	6	4	40	0.00025	0.177
SHD	0	2	2	2	20	0.000283	0.187

Supplementary Table S8 FDR-adjusted p-values for survival validation and biomarker analyses

Analysis Type	Cohort	Panel	Endpoint / Biomarker	Raw p-value	FDR-adjusted q-value
Survival	KIRC	Panel 1	OS	0.003	0.003
Survival	KIRC	Panel 2	OS	0.011	0.011
Survival	KIRC	Panel 3	OS	0.005	0.005
Survival	Pan-cancer	Panel 1	OS	1.83E-11	8.23E-11
Survival	Pan-cancer	Panel 2	OS	2.77E-11	8.32E-11
Survival	Pan-cancer	Panel 3	OS	2.20E-34	3.95E-33
Survival	KIRC	Panel 1	DSS	4.17E-4	5.36E-4
Survival	KIRC	Panel 2	DSS	2.51E-4	4.10E-4
Survival	KIRC	Panel 3	DSS	7.90E-4	9.48E-4
Survival	Pan-cancer	Panel 1	DSS	1.63E-10	4.20E-10
Survival	Pan-cancer	Panel 2	DSS	2.29E-11	8.23E-11
Survival	Pan-cancer	Panel 3	DSS	3.23E-29	1.94E-28
Survival	KIRC	Panel 1	PFS	3.26E-4	4.51E-4
Survival	KIRC	Panel 2	PFS	1.53E-4	2.76E-4
Survival	KIRC	Panel 3	PFS	3.05E-4	4.51E-4
Survival	Pan-cancer	Panel 1	PFS	1.05E-4	2.10E-4
Survival	Pan-cancer	Panel 2	PFS	2.40E-5	5.40E-5
Survival	Pan-cancer	Panel 3	PFS	8.76E-34	7.88E-33
Biomarker	TCGA	Panel 3	TMB	1.95E-293	7.81E-293
Biomarker	TCGA	Panel 3	MSI	4.65E-90	4.65E-90
Biomarker	TCGA	Panel 3	HRD	6.94E-231	1.39E-230
Biomarker	PAWG	Panel 3	TMB	8.45E-150	1.13E-149

Supplementary Table S9 Holm-adjusted p-values for panel variables in multivariate Cox models (within each endpoint–subgroup panel, m=3)

Endpoint	Subgroup	Panel	Raw p-value	Holm-adjusted p-value
OS	Localized	Panel 1	0.001	0.003
OS	Localized	Panel 2	0.007	0.014
OS	Localized	Panel 3	0.136	0.136
OS	G2/G3	Panel 1	0.003	0.009
OS	G2/G3	Panel 2	0.019	0.038
OS	G2/G3	Panel 3	0.097	0.097
OS	All	Panel 1	0.031	0.093
OS	All	Panel 2	0.194	0.388
OS	All	Panel 3	0.288	0.388
DSS	Localized	Panel 1	0.032	0.096
DSS	Localized	Panel 2	0.036	0.096
DSS	Localized	Panel 3	0.051	0.096
DSS	G2/G3	Panel 1	0.015	0.045
DSS	G2/G3	Panel 2	0.018	0.045
DSS	G2/G3	Panel 3	0.04	0.045
DSS	All	Panel 1	0.098	0.267
DSS	All	Panel 2	0.159	0.267
DSS	All	Panel 3	0.089	0.267
PFS	Localized	Panel 1	0.122	0.244
PFS	Localized	Panel 2	0.122	0.244
PFS	Localized	Panel 3	0.041	0.123
PFS	G2/G3	Panel 1	0.006	0.018
PFS	G2/G3	Panel 2	0.024	0.048
PFS	G2/G3	Panel 3	0.047	0.048
PFS	All	Panel 1	0.076	0.132
PFS	All	Panel 2	0.066	0.132
PFS	All	Panel 3	0.025	0.075

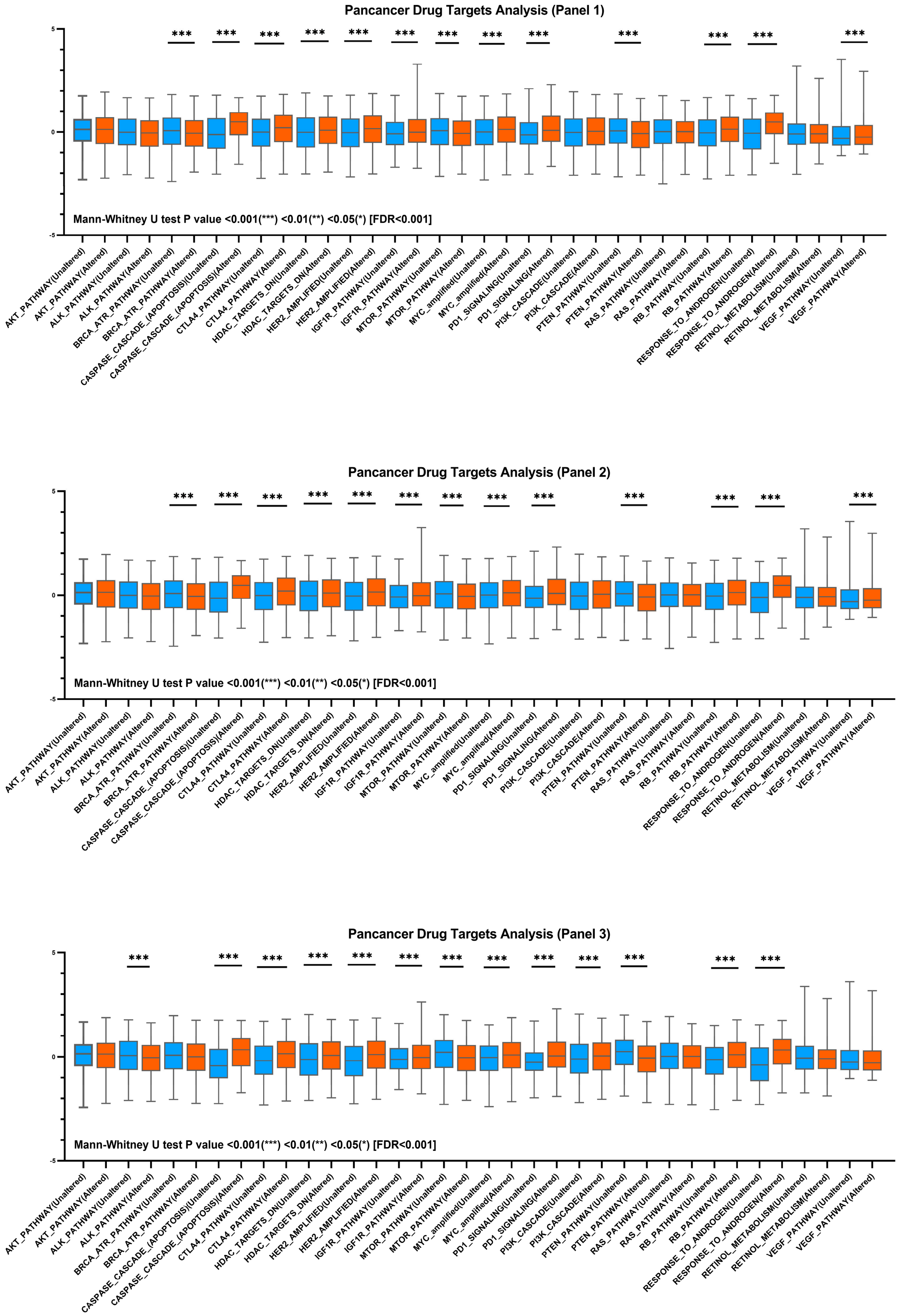

Supplementary Figure S1
Drug Targets Analysis

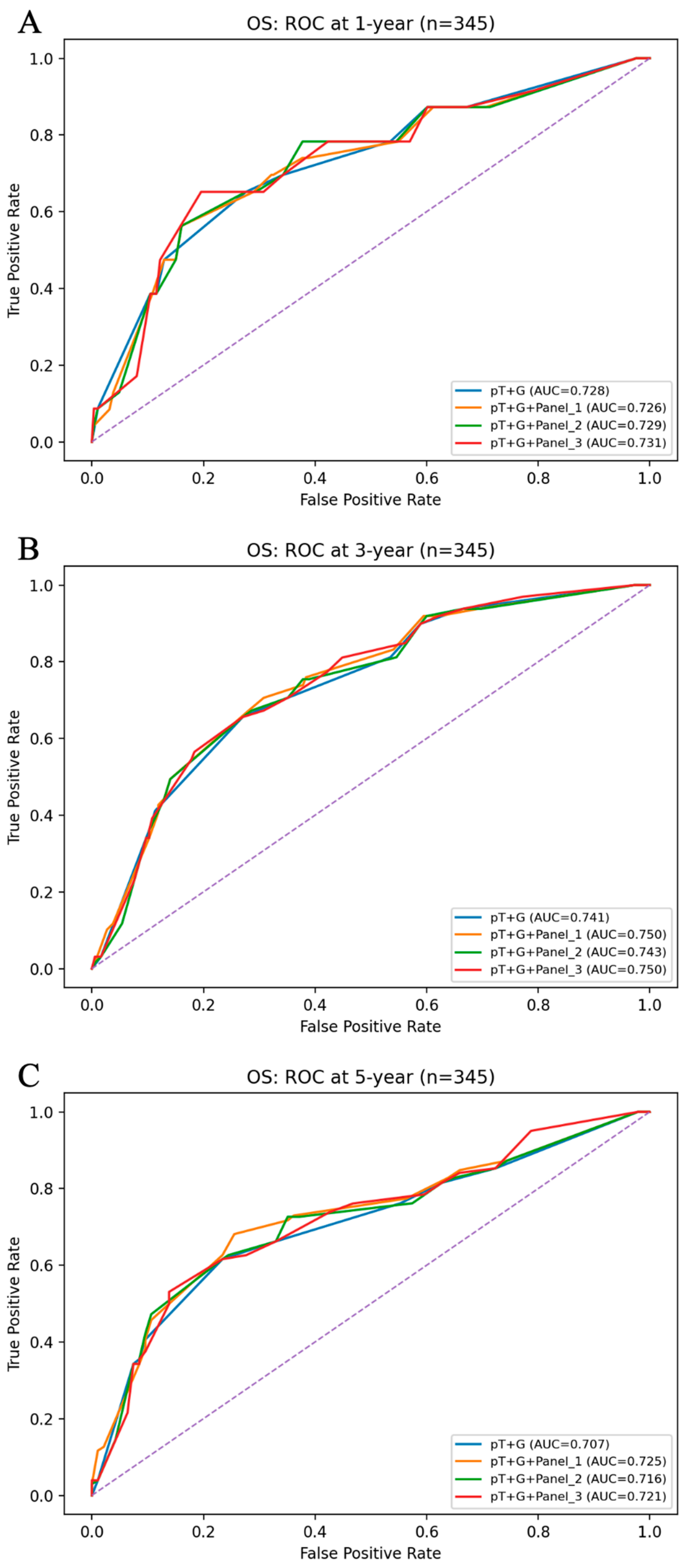

Supplementary Figure S2

Time-dependent ROC curves comparing tumor stage + grade (pT+G) with molecular panels at 1, 3, and 5 years for OS in the TCGA-KIRC cohort.

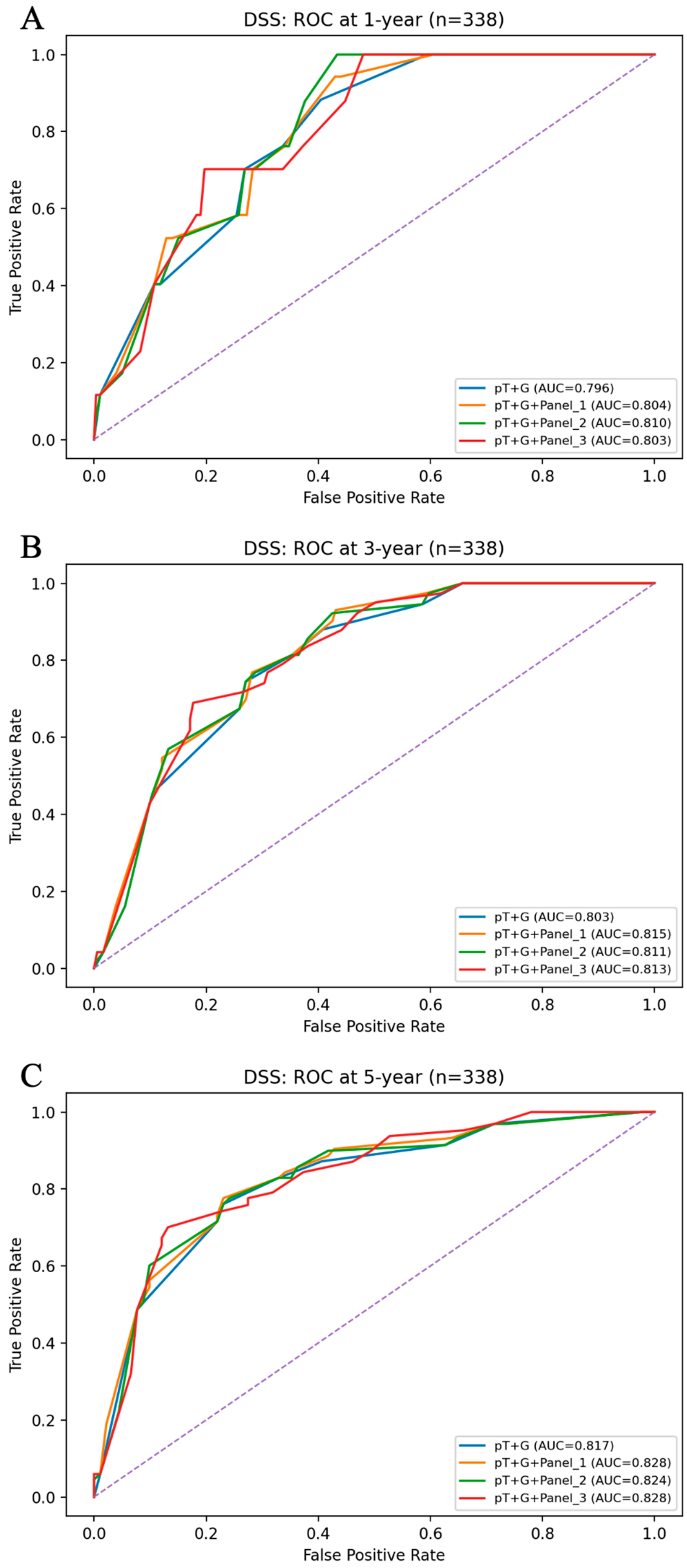

Supplementary Figure S3

Time-dependent ROC curves comparing tumor stage + grade (pT+G) with molecular panels at 1, 3, and 5 years for DSS in the TCGA-KIRC cohort.

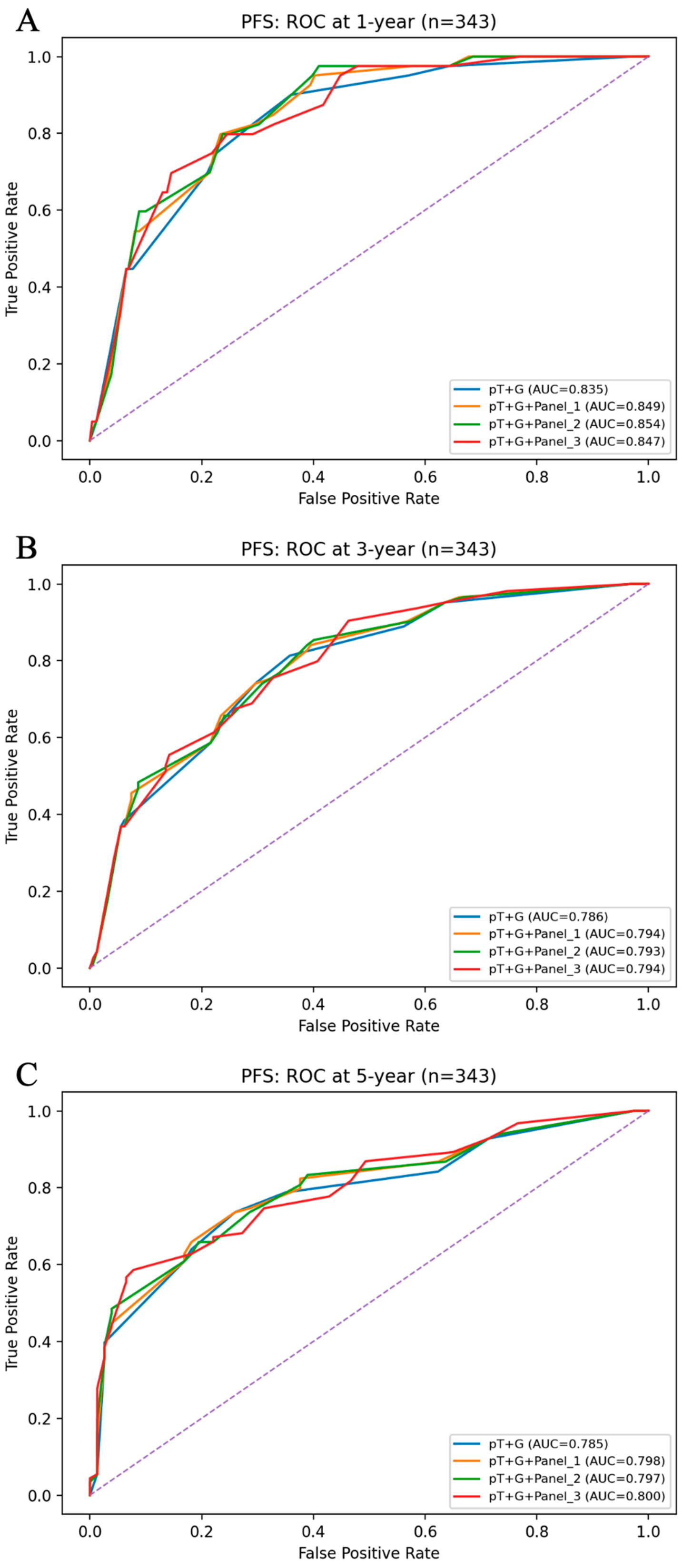

Supplementary Figure S4

Time-dependent ROC curves comparing tumor stage + grade (pT+G) with molecular panels at 1, 3, and 5 years for PFS in the TCGA-KIRC cohort.

Definition and Selection of SMGs, Driver Genes, and Predisposing Genes

Significantly mutated genes (SMGs) were defined as genes exhibiting a statistically higher mutation frequency in the high-risk group compared with the control group, based on frequency-based comparison adjusted for multiple testing.

Driver genes were identified as functionally validated oncogenes or tumor-suppressor genes annotated in established cancer genomics databases, including COSMIC (Catalogue of Somatic Mutations in Cancer), OncoKB, and the Cancer Gene Census.

Predisposing genes were defined as hereditary cancer-related genes curated from ClinVar and the Cancer Predisposition Gene Database, as well as those reported in key literature describing germline variants associated with tumor susceptibility.

References

1. Forbes SA, Beare D, Boutselakis H, et al. COSMIC: somatic cancer genetics at high-resolution. Nucleic Acids Res. 2017;45(D1):D777–D783. doi:10.1093/nar/gkw1121
2. Chakravarty D, Gao J, Phillips SM, et al. OncoKB: A Precision Oncology Knowledge Base. JCO Precis Oncol. 2017;2017:PO.17.00011. doi:10.1200/PO.17.00011
3. Sondka Z, Bamford S, Cole CG, Ward SA, Dunham I, Forbes SA. The COSMIC Cancer Gene Census: describing genetic dysfunction across all human cancers. Nat Rev Cancer. 2018;18(11):696–705. doi:10.1038/s41568-018-0060-1
4. Rahman N. Realizing the promise of cancer predisposition genes. Nature. 2014;505(7483):302–308. doi:10.1038/nature12981
5. Huang KL, Mashl RJ, Wu Y, et al. Pathogenic germline variants in 10,389 adult cancers. Cell. 2018;173(2):355–370.e14. doi:10.1016/j.cell.2018.03.039
